# Development of Thixotropic Molecular Oleogels Comprising Alkylanilide Gelators by Using a Mixing Strategy

**DOI:** 10.3390/gels9090717

**Published:** 2023-09-04

**Authors:** Yutaka Ohsedo

**Affiliations:** Division of Engineering, Faculty of Engineering, Nara Women’s University, Kitauoyahigashi-Machi, Nara 630-8506, Japan; ohsedo@cc.nara-wu.ac.jp

**Keywords:** alkylanilides, low-molecular-weight organogelators, mixed molecular gels, oleogels, thixotropic behavior

## Abstract

Molecular oleogels have the potential to be used as materials in healthcare applications. However, their design and synthesis are complex, thus requiring simple and effective methods for their preparation. This paper reports on alkylanilides that are low molecular-weight organogelators, which when appropriately mixed with different alkyl chain lengths could result in the formation of mixed molecular gels that exhibit excellent gel-forming ability and mechanical properties. In addition, the single and mixed molecular organogel systems were found to be applicable as single and mixed molecular oleogel systems capable of gelling oils such as olive oil and squalane. This has been found to be true, especially in molecular oleogel systems consisting of squalane, which is used as solvents in healthcare. The mixed squalene-molecular oleogel systems showed an increase in the critical (minimum) gelation concentration from 1.0 to 0.1 wt.% in the single system and an improvement in the thixotropic behavior recovery time. The thixotropic behavior of the molecular oleogels in the mixed system was quantitatively evaluated through dynamic viscoelasticity measurements; however, it was not observed for the single-system molecular oleogels. Scanning electron microscopy of the xerogels suggested that this behavior is related to the qualitative improvement of the network owing to the refinement of the mesh structure. These mixed molecular oleogels, composed of alkylanilides displaying such thixotropic behavior, could be used as candidates for ointment-base materials in the healthcare field.

## 1. Introduction

Low-molecular-weight gelators (LMWGs) form fibers from molecular aggregate formations based on the supramolecular interactions among their molecules, resulting in a macroscopic network of materials containing solvents as molecular gels [1,2,3,4]. These LMWG-derived molecular gels have gained significant research interests owing to their sensitive and adaptable responses at both the molecular and macroscopic levels, thus effectively utilizing the reversibility and selectivity of their supramolecular interactions in response to changes in the external stimuli [5,6,7,8,9,10,11,12,13,14,15,16,17,18]. Particularly, molecular gels with thixotropic properties, which can reversibly change from a gel state to a liquid sol under external mechanical forces [19,20], are of considerable research interest. These functional molecular gels can be employed in healthcare and medical fields as novel functional soft materials [21,22,23,24,25], distinct from conventional polymer gels. Therefore, new LMWGs must be developed; however, this requires considerable efforts. It is believed that the application of known substances such as gelators or the further improvement of the physical properties of known gelators by mixing may be useful as a new research direction. Various efforts have been made to improve the properties of mixed molecular gels by mixing existing gelators. A method for creating molecular gels using existing gelators involves the synthesis of new multicomponent molecular-gel systems by mixing several gelators and polymers [26,27,28,29,30,31,32,33,34,35,36]. In this research direction, the author determined that molecular gels obtained by mixing LMWGs, such as alkylhydrazides [37], alkylureas [38], alkylamides [39,40], alkyl-*D*-glucamines [41] and alkylhydantoins [42], with different alkyl chains exhibit thixotropic properties not exhibited by single systems. Such a property is considered necessary in medical applications of molecular gels, including ointment-base materials and injectable pharmaceutical-base materials [21,22,23,24,25]. These mixed molecular organogel systems that exhibit good mechanical properties, namely thixotropic properties required for paintable applications, may also find applicability in oleogel systems, which are under active investigation and research. Oleogel systems use naturally derived oils as solvents, which are low in toxicity to the human body, nutritious and have a long history of use in the human body. Together with organogel systems that use organic solvents and hydrogel systems using water solvents, they have been examined for development across a variety of basic applications, including healthcare, medical and even food applications, including the development of oleogelators [43,44,45]. The development of oleogelators, together with organogel systems using organic solvents and hydrogel systems using water-soluble solvents, has been investigated for a variety of basic applications, including healthcare, medical and food applications. Recently, studies on drug delivery applications using oleogels have also been actively conducted [46]. It is expected that the development of new high-performance oleogelators that meet the performance requirements of various applications and the expansion of new applications using these oleogelators will continue to grow.

This study investigated the creation of mixed molecular gels using alkylanilides as LMWGs, in which the nitrogen–hydrogen moiety of alkylamides is modified to a nitrogen–phenyl group. Anilide compounds (phenyl amide derivatives) are known as the active moiety of organic compounds such as carboxin and oxycarboxin, which are effective fungicides [47], and are important compounds for which various synthetic studies and derivative synthesis have been conducted. For example, the synthesis and bioassay assisted by molecular dynamics simulations for carboxin was carried out [48], and the synthesis of oxycarboxin derivative using 2-(benzylsulfonyl)benzothiazole unit and their antifungal activity has been reported [49]. Many of the LMWGs investigated so far have been found to comprise phenyl groups, which are presumed to play a role in regulating the interactions among molecules, such as their bulkiness and π–π interaction [1,2,3,4]. As for the molecular gel studies of alkylanilides, several studies have reported on the gel-forming ability of LMWGs containing anilide moieties and some alkylanilides [50,51]. In addition, the significant effect of the solvent isomer on the thermodynamic properties and the morphology of the LMWG containing alkylanilide moieties and the creation of a hybrid gel with this organogelator and polymer were reported [52]. Although there are prior reports of LMWGs containing anilide or alkylanilide moieties as shown above, they have not yet been studied in single or mixed molecular gels of “alkylanilides” rather than alkylanilide derivatives.

This paper reports on the gel-forming ability and properties of the molecular gels of three alkylanilides (C18, C16, and C14) with different alkyl chain lengths in single and mixed systems. Additionally, the performance improvement of the gels is studied by mixing them, especially with respect to their thixotropy behavior, which is an important aspect of ointment-base materials in medical applications. In particular, the thixotropic behavior of mixed oleogels using squalane as a solvent [53], which is useful in healthcare and cosmetic applications, was evaluated and investigated.

## 2. Results and Discussion

**Figure 1:**
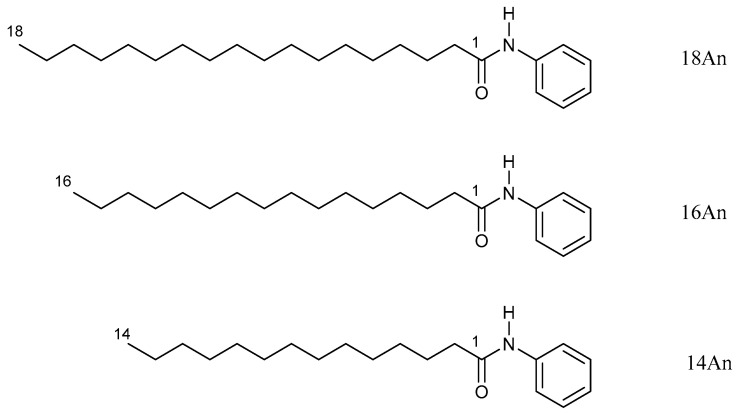
Chemical structures for the alkylanilides.

**Figure 2:**
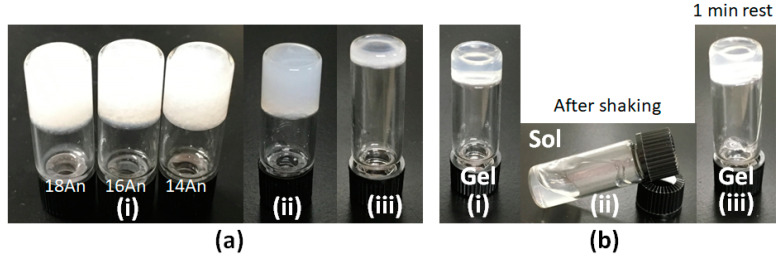
(**a**) Photographs of squalane oleogels at 1.0 wt.%: (i) single-component oleogel, (ii) two-component oleogel (**18An**/**16An** 1/1), (iii) three-component oleogel (**18An**/**16An**/**14An** 1/1/1). (**b**) Thixotropic behavior of **18An**/**16An**/**14An** 1/1/1 (0.25 wt.%) squalane oleogels: (i) gel before shaking, (ii) sol after shaking, and (iii) recovered gel after 1 min resting of sol.

The gel-forming properties and behavior of the gels composed of alkylanilide homologues with different alkyl chain lengths was evaluated by preparing samples of single and mixed molecular gels, and the effect of mixing on the mechanical properties of the gels was investigated and studied. In this study, alkylanilides (Figure 1), such as stearanilide (**18An**), palmitanilide (**16An**), and myristanilide (**14An**), were employed to investigate their critical gel concentrations (CGC) in various organic solvents (Table 1, all resulting in opaque gels). Based on the findings, *n*-octane and squalane were identified as suitable solvents for forming alkylanilide gels in terms of gel-forming ability (low CGC). Moreover, alkylanilides with longer alkyl chains exhibited enhanced gel-forming abilities in terms of CGC. As shown in alkylamide-based organogels, the longer the alkyl chain length, the greater the alkyl–alkyl interaction, which is thought to favor crystal formation leading to gel formation [39]. For solvents with polar groups, CGC tends to be higher, perhaps because intermolecular hydrogen bonds are inhibited, but propylene carbonate seems to be an exception. Compared to alkylamides [39], the presence of the phenyl group in alkylanilides increased their solubility in polar solvents such as DMF and tetrahydrofuran, resulting in increased CGC, while no significant differences were observed in other solvents. These compounds demonstrated superior organogelation properties when used in oleogels, specifically with olive oil and squalane as solvents, compared to alkylamide organogel systems [39]. The low CGC obtained with oleogels with olive oil and squalane may be because these nonpolar solvents are more prone to hydrogen bonding interactions even at low concentrations, as shown for alkylamide systems [39]. In particular, for further exploration of mixed oleogel systems, this study focused on the alkylanilides/squalane system because of its favorable oleogel-forming characteristics in CGC for healthcare applications. The mixed squalane–oleogel systems were exclusively examined using various combinations of alkylanilides in squalane (Table 2). The examination of binary and tertiary alkylanilides/squalane systems revealed that mixed gels exhibited improved CGC values compared to single gels, from 1.0 wt.% to 0.1 wt.%. No differences were observed among the CGC values of different component ratios; however, the comparison of the single and mixed systems at the same concentrations revealed the gel to be opaque for the former and translucent for the latter, indicating a qualitative improvement (Figure 2a). Additionally, the mixed molecular-gel system demonstrates thixotropic behavior (Figure 2b), which is not observed in the single molecular-gel system (no recovery to the single molecular-gel system from its sol was observed after waiting one day). Furthermore, as shown in Table 2, the recovery from sol to gel in the thixotropic evaluation was shorter in the **18An**/**16An** and **18An**/**14An** systems, possessing a higher ratio of **18An**, and in the three-component system. Thus, the thixotropic behavior of alkylanilides was found to be better in mixed molecular gels obtained by mixing single systems, both two-component and three-component alkylanilide systems. All single and mixed gels displayed semi-transparent characteristics and maintained their gel states for a minimum of 6 months without macroscopic crystallization of alkylanilides visible to the naked eye.

**Figure 3:**
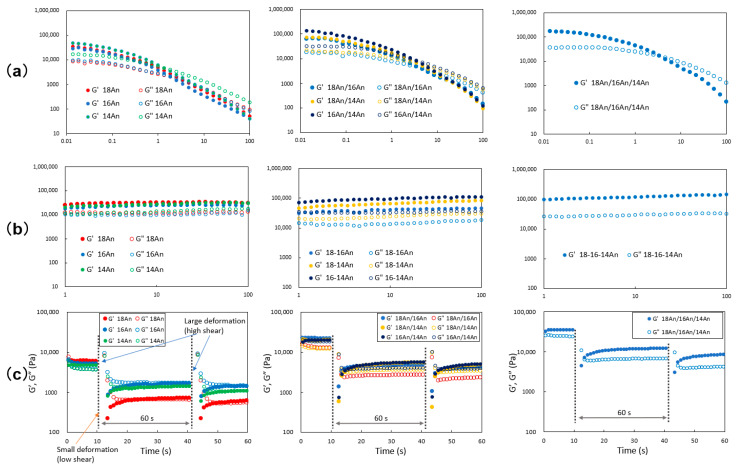
(**a**) Dynamic rheological properties of the mixed molecular alkylanilide oleogels (3.0 wt.% in squalane) with respect to frequency sweep. (**b**) Dynamic rheological properties of the mixed molecular squalane alkylanilide oleogels (3.0 wt.% in squalane) with respect to strain sweep. (**c**) Thixotropic behavior of **18An**/**16An**/**14An** 1/1/1 (0.25 wt.%) squalane oleogels: gel before shaking, sol after shaking, and recovered gel after 1 min resting of the sol.

The gel state and its thixotropic behavior were quantitatively evaluated using dynamic viscoelasticity measurements (Figure 3). Frequency dispersion measurements of the mixed gel samples show that the state of G′ (storage modulus) > G″ (loss modulus) is almost constant over a three-digit frequency range (1–100 rad s^−1^), and the values of G′ and G″ are independent of frequency characteristics, indicating a gel state similar to that exhibited by molecular gels (Figure 3a) [54]. It was observed that the height of the elastic modulus of the gel and the magnitude between G′ and G″ increased as the system went from a single component to a mixture and from two to three components (Figure 3a). Thus, it was shown that the gel became stiffer, and the gel state became more stable as it went from a single component to a mixture and from a two-component to a three-component system. Furthermore, strain dispersion measurements showed a transition from G′ > G″ (the gel state) to G′ < G″ (the sol state), indicating that the gel state is present at small strains in this measurement (Figure 3b). The strain values for this transition increased with mixing compared to the single system (Figure 3b). Thus, it was found that the elastic modulus of the gels increased as the gels were mixed and as the gels became three-component systems instead of two-component systems, forming mechanically stable gels that require a large amount of strain at the transition. In addition, rheological measurements performed on both single and mixed molecular gels showed that G″ exceeded G′ over a wide frequency range, providing evidence of polymer gel-like behavior of the mixed alkylanilide molecular gel [55].

The thixotropic behavior with respect to rheometry was evaluated by looking at the transitional changes in G′ and G″ of the gel samples after periodically applying large deformation shear (Figure 3c). According to the results, the recovery from the sol state to the gel state after the change from the gel state to the sol state after applying a large deformation shear was greater for the mixed gel than for the single gel. Furthermore, the behavior of elastic modulus recovery from G′ < G″ (sol state) to G′ > G″ (gel state) immediately after applying large deformation shear to the gel was observed to be steeper in the mixed gel. However, unlike the macroscopic and long-term recovery phenomenon as shown in Figure 2b for the mixed gel in the vial tube, the rheometry evaluation results showed that the recovery behavior of the mixed gel system was insufficient (did not completely return to the elastic modulus value before fracture) even after repeated short-time periodic fracture-recovery cycles, and a decrease in the elastic modulus of the recovered gel was observed with each repeated fracture. Further study of mixing ratios and the addition of other ingredients is considered necessary to obtain higher-performance thixotropic oleogels. A decrease in the elastic modulus of the recovered gel was observed with each repeated fracture. However, in the single system, the recovered gel after fracture showed G′–G″, and there was significant separation of G′ and G″ as shown in the mixed system. The thixotropic properties of the single system gels were assessed in vials, and no thixotropic properties were observed, but the results in Figure 3c show that the single system gels also appear to be thixotropic. This may indicate that although there is no macroscopic thixotropy that can be visually observed in the single system gels, gel recovery occurs in the 0.1 mm scale micro-region that can be evaluated by rheometry. Furthermore, among the mixed systems, the separation of G′ and G″ was larger, and the recovery behavior was better for the three-component molecular gels than for the two-component systems. Compared to the mixed alkylamide squalane gel [40], the mixed alkylanilide gel shows better recovery from sol to gel after gel breakdown and a more stable plot. This may be due to the increased intermolecular interactions caused by introducing phenyl groups, which may favor the formation of fibers in the gel network after sol formation. Thus, the thixotropic behavior of alkylanilides was quantitatively found to be better in the two-component and even three-component molecular gel systems than in the single system by dynamic viscoelasticity evaluation using rheometry.

**Figure 4:**
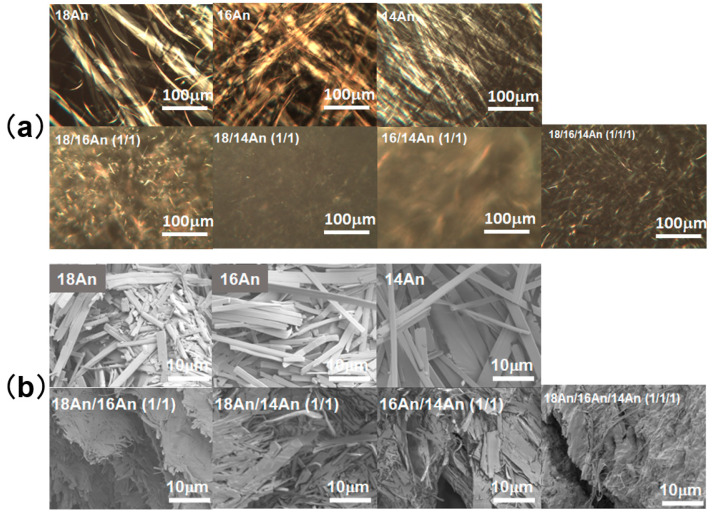
(**a**) POM images of the single and mixed molecular alkylanilide oleogels (solvent: squalane) under the crossed nicols. (**b**) SEM images of the single and mixed alkylanilide xerogels obtained from molecular gels at the concentrations of CGC with *n*-octane as the solvent.

In order to investigate the internal structure, specifically the internal network structure, of the mixed molecular oleogels obtained by mixing, the surface topography of the gel components was analyzed by polarized optical microscopy (POM). As a result, it was found that the xerogel consists of a network of fibrous crystals as components since the xerogel is composed of fiber-like materials with a width of several tens of μm and a length of 100 μm, anisotropically oriented (Figure 4a). Such network structures and surface morphologies of the constituents are commonly observed in various gel systems [1,2,3,4]. In scanning electron microscopy (SEM) measurement, however, the mixed system showed a network composed of finer components than the single system (Figure 4b). As evident from the SEM images, in the mixed system, tape-like fibers with a width of micrometers were observed, whereas sheet-like fibers with a width of tens of micrometers were observed in the single system. This is similar to the trend observed in the mixed molecular gel system [37,38,39,40,41,42]. Such changes in the gel network structure are characterized by changes in the microstructural components, potentially resulting in network densification. This densification of the network structure in mixed systems may lead to (1) an increase in the ability of the gel to form a network of gels due to the miniaturization of the network components (it may lead to improved CGC) and (2) an increase in the transparency of the gel due to the suppression of scattering of visible light by the miniaturization of the network components, as supported by previous studies [35,36,37,38,39,40,41,42]. The improved mechanical properties of the mixed oleogels (increasing strain changing from gel to sol in the mixed system in Figure 3a) and their ability to recover from the sol state to the gel state by the application of external forces (thixotropic behavior) are also presumed to contribute to the finer network components and the associated densification of the network structure. The reason why the thixotropic properties were better with a higher ratio of **18An** can be attributed to two major factors: (1) the miniaturization of the components of the gel network by mixing resulted in a qualitative improvement of the network (this tendency is greater in three-component systems than in two-component systems) and (2) the longer alkyl chain length is more favorable for recovery from sol to gel due to greater intermolecular interactions. The decrease in the elastic modulus in the gel recovered from the sol in the thixotropic evaluation results (Figure 3c) may also indicate that the gel mesh has not fully recovered. In addition, the result that the trend in the magnitude of recovery results in a three-component system > two-component system > one-component system may indicate that it is more difficult to recover a crystal once it has been destroyed, considering that the size of the components (crystals) in the network increases in this order.

**Figure 5:**
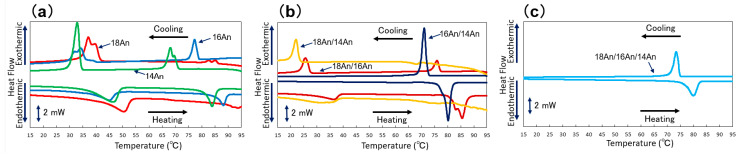
DSC curves of the mixed alkylanilide oleogels. All samples are 3 wt.% squalane gels and mixed in a molar ratio (heating and cooling rate is 10 °C/min): (**a**) single and binary systems, (**b**) binary systems, (**c**) tertiary systems.

To understand the network formation of LMWGs in the gel state, thermal differential scanning calorimetry (DSC) was performed to analyze the gels. In this DSC measurement, the thermal properties of the gel and sol states are assessed by looking at the heat input/output and their transition temperatures as the gel sample undergoes a gel-to-sol transition through a temperature increase process, followed by a sol-to-gel transition through a temperature decrease process. Figure 5 and Table 3 illustrate the corresponding peaks and Δ*H* changes observed during both temperature increase and decrease processes. These peaks signify the gel melting ability with the increase in temperature and gel formation with the decrease in temperature, as shown in general gelator systems [1]. However, unlike previous studies on LMWGs that demonstrated a peak shift toward lower temperatures due to the mixing [37,38,39,40,41,42], thus supporting network structure refinement; in this study, such a distinct trend was not observed for any of the alkylanilide samples. This suggests that the inclusion of the phenyl group in the alkylanilides might introduce complex molecular interactions that hinder the melting of the gel fibers. Furthermore, these results imply the presence of complex thermal behaviors that differ from those exhibited by simple alkylamides and their mixed molecular gels. However, it can be seen that the mixed system oleogels show different thermal behavior than the single system oleogels, corresponding to the differences in the macroscopic gel state obtained visually, the differences in mechanical properties obtained from dynamic viscoelasticity measurements, and the differences in the shape of the components obtained from the SEM images obtained, in the single and mixed systems.

**Figure 6:**
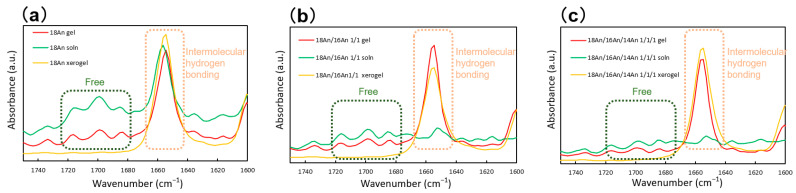
ATR–FTIR spectra of the alkylanilides in different states (a gel, squalane solution, and xerogel) in the carbonyl stretching region: (**a**) single-system **18An** (solution: 0.5 wt.%), (**b**) binary-system **18An**/**14An** 1/1 (solution: 0.01 wt.%), and (**c**) tertiary-system **18An**/**16An**/**14An** 1/1/1 (solution: 0.01 wt.%).

To understand the role of hydrogen bonds in the gel, solution, and xerogel states among the gelators, the attenuated total reflectance Fourier-transform infrared (ATR–FTIR) spectroscopy absorption spectra of the three states were compared. Figure 6 illustrates the ATR–FTIR spectra of the absorption bands related to carbonyl groups involved in hydrogen bonding. In the single-component system, the absorption band corresponding to the carbonyl group derived from intermolecular hydrogen bonding (approximately 1640–1680 cm^−1^) exhibited a larger intensity than the absorption band of the free carbonyl group (approximately 1680–1720 cm^−1^) with increasing gelator concentration, i.e., in the order of solution, gel, and xerogel states (similar trends were observed for **16An** and **14An**). Notably, this trend was even more pronounced in the mixed system. Moreover, in both the single and mixed systems, the absorption bands for the free-derived carbonyl groups as well as the intermolecular hydrogen-bond-derived carbonyl groups were prominent. Similar trends were observed in other single- and two-component systems. No significant differences in absorption spectra in other regions were observed. These findings suggest that the fiber formation through hydrogen bonding might be stronger in the mixed system than that in the single system. This enhanced ease in the formation of intermolecular hydrogen bonds in the mixed systems could explain the favorable recovery of fibers from the sol state to the gel state after the gel network undergoes disruption due to external mechanical forces. The outcomes and patterns observed in this study align with those reported for other mixed-system molecular gels [37,38,39,40,41,42].

## 3. Conclusions

This study demonstrated the gel-forming properties and mechanical characteristics of alkylanilides as LMWGs for mixed molecular oleogels. The mixing strategy proved effective in enhancing gel formation and mechanical properties of gels compared to single-gel systems. Evidently, alkylanilides represent novel LMWGs capable of gelling hydrophobic organic solvents. Moreover, the mixed squalane–oleogel systems exhibited improved thixotropic behavior, particularly in terms of mechanical properties, despite each single molecular oleogel displaying poor thixotropic behavior, as indicated by the rheometric results. These findings suggest the possibility of utilizing alkylanilides as additives in new mixed molecular oleogels and as potential candidates for the base materials in drug-release ointments. These novel, mixed molecular oleogels, characterized by their thixotropic behavior, could be used as ointment-base materials in the healthcare and cosmetic industries, given that they form gels of squalane oil—an ingredient commonly used in healthcare and cosmetic products. For further improving molecular oleogels, investigations are warranted to elucidate the principles of suitable gelators, and methods are required to enhance mechanical properties through mixing. Additionally, exploring the correlation between molecular structure and physical properties would contribute to the development of more effective and versatile molecular oleogels.

## 4. Materials and Methods

Alkylanilides with different alkyl chain length, stearanilide (*N*-phenylstearamide, 98%), palmitanilide (*N*-phenylpalmitamide, 98%), and myristanilide (*N*-phenylmyristamide, 99%), were purchased from Tokyo Chemical Industry Co., Ltd., Tokyo, Japan. All other chemicals were purchased from Wako Pure Chemical Industries, Ltd., Tokyo, Japan, and used without further purification.

The gelation process for alkylanilides was conducted as follows: alkylanilide crystals were placed in a vial containing a solvent with a predetermined concentration (wt.%), and the vial was sealed. The vial was then heated in a dry bath at 100 °C to facilitate the dissolution of alkylanilide crystals. The resulting solution was allowed to cool to room temperature and stand for 30 min to assess gelation. The formation of mixed molecular gels was achieved by combining alkylanilide homologues in integer molar ratios, as detailed in the main text.

The gelation ability and thixotropic behavior were evaluated using the vial inversion method, wherein a vial containing the gel was inverted and determined to be in a gel state if it did not drip. Thixotropic behavior was assessed by observing whether the gel recovered from a sol state to a gel state after being broken by vortexing, left to stand for a specific period, and inverted (if it did not recover and remained a sol, it would drip).

Polarized optical microscopy (POM) observations of the squalane oleogels were performed using a Leica DM2500 polarized optical microscope (Leica Microsystems GmbH, Wetzlar, Germany) equipped with crossed nicols.

SEM image measurements were conducted using a SU-8000 scanning electron microscope (Hitachi High-Technologies Corporation, Tokyo, Japan) operating at 1.0 kV. The vacuum-dried organogels (xerogels), prepared with *n*-octane as the solvent (xerogels), were measured after one day of drying at 25 °C and an additional day of drying at 80 °C in vacuum. The samples were placed on a conductive tape on the brass SEM stage. Prior to imaging, the samples were coated with a 10 nm thick layer of Pt using a sputtering technique to enhance electrical conductivity.

Dynamic rheological measurements were carried out by use of an MCR-301 rheometer (Anton Paar Japan K.K., Tokyo, Japan) equipped with an 8 mm diameter parallel plate set at a gap of 0.50 mm. Frequency sweeps were performed with a strain amplitude (γ) of 0.01%. Strain sweeps were conducted at a constant angular frequency of 1 rad s^−1^. The repeated step-shear measurements involved applying a small strain with an amplitude of 0.01% and a frequency of 1 Hz, followed by a large strain with a shear rate of 3000 s^−1^ for 0.1 s.

Thermal analysis of the oleogels was carried out using an EXSTAR6220 differential scanning calorimeter (DSC) (Hitachi High-Tech Corporation, Tokyo, Japan) equipped with a closable sample pan made of Ag.

ATR–FTIR spectra were recorded using a Cary 670 FTIR spectrometer (Agilent Technologies Japan, Ltd., Tokyo, Japan) coupled with a single bounce diamond attenuated total reflectance (ATR) accessory.

## Figures and Tables

**Table 1:** Critical gel concentrations (CGC, wt.%) of alkylanilides in organic solvents.

Solvent	18An	16An	14An
Propylene carbonate	2	2	2
*N*,*N*-Dimethyl formamide	6	7	S ^1^
Methanol	5	5	6
Ethanol	3	2	7
1-Butanol	4	8	S ^1^
Dichloroethane	3	3	4
Tetrahydrofuran	10	S ^1^	S ^1^
Ethyl acetate	3	3	4
Toluene	2	2	4
*n*-Octane	1	1	1
Olive oil	3	3	4
Squalane	1	1	1

^1^ S: solution at 10 wt.%.

**Table 2:** Gelator concentration (wt.%) for a 1 min recovery time from sol to gel in thixotropic evaluation using the inversion method in squalane oleogels.

	Mixed Squalane—Oleogel Samples ^1^			
Mixed Molar Ratio	18An/16An	18An/14An	16An/14An	18An/16An/14An
5/1	0.25 (0.10) ^2^	0.50 (0.10) ^2^	0.25 (0.10) ^2^	
2/1	0.25 (0.10) ^2^	0.50 (0.10) ^2^	0.25 (0.10) ^2^	
1/1	0.50 (0.10) ^2^	0.50 (0.10) ^2^	0.50 (0.10) ^2^	
1/2	1.00 (0.10) ^2^	0.50 (0.10) ^2^	0.50 (0.10) ^2^	
1/5	1.00 (0.10) ^2^	0.50 (0.10) ^2^	1.00 (0.10) ^2^	
1/1/1				0.25 (0.10) ^2^

^1^ Data are shown for the total wt.% of each component. ^2^ The value in the parenthesis represents CGC at the composition.

**Table 3:** Transition temperatures of single and mixed alkylanilide oleogels obtained through differential scanning calorimetry (DSC) measurements (heating and cooling rate is 10 °C/min).

Samples ^1^	T_gel to sol_ on Heating/°C (ΔH/mJ mg^−1^)	T_sol to gel_ on Cooling/°C (ΔH/mJ mg^−1^)
**18An**	43.0 (13.4), 89.7 (1.2)	41.4 (13.9), 86.5 (1.3)
**16An**	37.1 (5.7), 84.2 (3.2)	79.5 (3.2)
**14An**	36.9 (11.1), 81.1 (5.7)	33.9 (11.1), 71.2 (5.7)
**18An**/**16An** 1/1	30.7 (4.4), 81.4 (7.9)	27.2 (4.0), 76.9 (2.2)
**18An**/**14An** 1/1	22.9 (5.7)	23.1 (5.6)
**16An**/**14An** 1/1	77.9 (8.3)	71.7 (8.1)
**18An**/**16An**/**14An** 1/1/1	76.4 (7.3)	75.2 (7.4)

^1^ All samples are 3 wt.% squalane gels and mixed in molar ratios. Transition temperatures are defined as the onset temperature of the peaks of DSC curves.

## Data Availability

Not applicable.
